# Correlation between vascular stenosis severity and dizziness symptoms and neurological prognosis in elderly patients with acute ischemic stroke

**DOI:** 10.3389/fneur.2025.1730875

**Published:** 2026-01-12

**Authors:** Yu Liu, Wei Li, Weijie Zhao, Yiyao Song, Junting Huo

**Affiliations:** Department of Neurology, Affiliated Chuiyangliu Hospital of Tsinghua University, Beijing, China

**Keywords:** acute ischemic stroke, dizziness, elderly patients, prognosis, vascular stenosis

## Abstract

**Background:**

Dizziness is a frequent complaint among elderly patients with acute ischemic stroke. However, its association with different grades and locations of arterial stenosis remains unclear. This study aimed to assess the links between stenosis severity, posterior circulation involvement, dizziness, and short-term neurological outcomes.

**Methods:**

A retrospective analysis was performed on 134 elderly patients with acute ischemic stroke admitted from January 2024 to May 2025. All patients underwent Computed Tomography Angiography (CTA) or MRA of the intracranial and extracranial arteries, including the common and internal carotid arteries, the middle cerebral artery, the vertebral artery (VA), and the basilar artery (BA). Stenosis of extracranial segments was measured with North American Symptomatic Carotid Endarterectomy Trial (NASCET) criteria, while intracranial segments were assessed using Warfarin–Aspirin Symptomatic Intracranial Disease Trial (WASID) standards. Patients were grouped by the most severely affected major supplying artery as mild (<50%), moderate (50–69%), or severe (≥70% or occlusion) stenosis. A separate vertebrobasilar artery (VA/BA) stenosis group included individuals with ≥50% stenosis of the vertebral or BA, regardless of anterior circulation status. Collected data included baseline characteristics, dizziness occurrence, Dizziness Handicap Inventory (DHI) scores, admission National Institutes of Health Stroke Scale (NIHSS) scores, and 3-month modified Rankin Scale (mRS) scores. Univariate and multivariate logistic regression analyses were conducted to identify risk factors.

**Results:**

The severe stenosis group and the VA/BA stenosis group showed higher dizziness rates than the other groups (44.9 and 50.0%, *p* < 0.01). Cerebellar and brainstem infarctions were more frequent in patients with VA/BA stenosis (83.3%), and these infarcts also appeared more often in the dizziness group (65.8% vs. 19.8%). Multivariate analysis indicated that VA/BA stenosis (OR = 3.42, 95% CI: 1.28–9.13) and posterior circulation infarction (OR = 4.51, 95% CI: 2.01–10.13) were independent factors related to dizziness. Severe stenosis (OR = 4.96) and VA/BA stenosis (OR = 3.18) were independently associated with functional dependence at 3 months (mRS ≥ 3). Admission NIHSS (OR = 1.42) and age (OR = 1.10) also suggested poorer outcomes.

**Conclusion:**

In elderly patients with acute ischemic stroke, severe arterial stenosis and VA/BA stenosis were linked to higher risks of dizziness and 3-month functional dependence. Posterior circulation infarction markedly increased the likelihood of dizziness. Enhanced vascular imaging assessment and attention to symptoms such as dizziness may help identify high-risk individuals and support personalized management.

## 1 Introduction

Acute ischemic stroke is a leading cause of disability and death in the elderly, with its incidence and disability rate rising significantly with age (1, 2). After stroke, patients often develop various neurological deficits that impair quality of life; among them, dizziness is both common and significantly affects rehabilitation compliance (3, 4). Because vertigo, disequilibrium, and nonspecific dizziness frequently overlap in elderly stroke patients, these symptoms were analyzed collectively as “dizziness” in this study. Nevertheless, careful bedside differentiation was performed to document their clinical features. In recent years, the impact of cerebral vascular stenosis on the prognosis of acute ischemic stroke patients has received growing attention (5). Shen’s et al. (6) study reported that patients with severe intracranial arterial stenosis have a 3-year stroke recurrence rate as high as 23%. Additionally, the trial by Kam et al. (7) demonstrated that atherosclerotic stenosis is associated with stroke recurrence and mortality (8). However, these studies mainly focused on “hard” endpoints like recurrence and death, while there is little discussion on subjective symptoms such as dizziness or their relationship with functional recovery. In particular, the effect of vertebrobasilar artery (VA/BA) stenosis on balance, cognition, and daily living abilities in the elderly has not been fully explored.

For dizziness-related symptoms, Lan et al. (9) conducted a retrospective analysis and found that the proportion of dizziness, vertigo, and gait instability in VA/BA stenosis patients is higher than in anterior circulation stroke patients. Most large-scale studies are based on Western populations, and few have systematically assessed the relationship between dizziness and neurological recovery. Regarding the impact of posterior circulation stenosis on daily living and cognition, Mosconi and Paciaroni (10) found in a prospective cohort that VA/BA stenosis-related stroke patients had higher mRS scores and poorer functional recovery. There is still a lack of systematic analysis in the Chinese population (11), which limits precise risk stratification and individualized management. Therefore, this study uses a retrospective cohort to systematically assess the relationship between cerebral artery stenosis severity and site, dizziness symptoms, and 3-month neurological outcomes in elderly patients with acute ischemic stroke, aiming to clarify the main risk factors and provide theoretical and practical reference for individualized clinical management.

## 2 Methods

### 2.1 Data collection

This is a single-center, retrospective cohort study. A total of 134 patients diagnosed with acute ischemic stroke were admitted to the neurology department of a tertiary hospital between January 2024 and May 2025. All patients received confirmed diagnoses from two neurologists with associate chief physician qualifications or higher, based on the latest Chinese Guidelines for the Diagnosis and Treatment of Acute Ischemic Stroke and the American Heart Association/American Stroke Association diagnostic criteria. The diagnostic basis included sudden focal neurological deficits lasting at least 24 h, with neuroimaging used to exclude intracranial hemorrhage and other non-ischemic conditions. Diffusion-weighted imaging (DWI) served as the preferred confirmation method whenever available. For patients who could not undergo DWI due to clinical or equipment limitations, diagnosis was made by combining clinical findings with a head Computed Tomography (CT) or routine Magnetic Resonance Imaging (MRI) after ruling out hemorrhagic lesions. Patients received cranial Computed Tomography Angiography (CTA) or Magnetic Resonance Angiography (MRA) within 72 h of admission and carotid ultrasound within 48 h.

Inclusion criteria: (1) Age ≥ 60 years; (2) Definite diagnosis of acute ischemic stroke, with CTA or MRA performed within 72 h of onset (patients unable to complete the exam due to allergy or renal dysfunction were excluded); (3) Completed carotid ultrasound at admission with clear stenosis and plaque data; (4) Complete inpatient medical records and evaluation data at 1 and 3 months after discharge were available.

Exclusion criteria: (1) Dizziness excluded as non-vascular (e.g., otogenic, drug-induced) by ENT or neurology; (2) History of moderate–severe cognitive impairment, aphasia, or severe communication disorder, making it impossible to assess subjective symptoms or scales; (3) Missing critical records or imaging; (4) TIA. All cases were approved by the ethics committee and gave informed consent.

### 2.2 Stenosis assessment and grouping

Cranial CTA or MRA images were interpreted independently by two radiologists with attending or higher qualifications; discrepancies were resolved by a third senior radiologist. If CTA and MRA results were inconsistent, CTA was used.

The assessment covered intracranial arteries (intracranial internal carotid artery, middle cerebral artery, VA, VB) and extracranial arteries (common carotid artery and extracranial internal carotid artery). Extracranial stenosis was measured with North American Symptomatic Carotid Endarterectomy Trial (NASCET) criteria: stenosis rate = (1 − residual lumen diameter ÷ distal normal lumen diameter) × 100%. Intracranial stenosis was calculated using the Warfarin–Aspirin Symptomatic Intracranial Disease Trial (WASID) method: stenosis rate = (1 − minimal lumen diameter ÷ proximal normal lumen diameter) × 100%. To ensure mutually exclusive grouping, the final classification was based on the artery with the highest degree of stenosis among all supplying vessels. Patients were assigned to four groups: (1) Mild stenosis: <50%, *n* = 39; (2) Moderate: 50–69%, *n* = 46; (3) Severe: ≥70% or occlusion, *n* = 49. Intracranial (MCA, intracranial ICA, VA, BA) and extracranial (CCA, extracranial ICA) arteries were assessed; (4) VA/BA stenosis group: patients with ≥50% stenosis of the vertebral artery (VA) or BA, regardless of stenosis in the anterior circulation. This grouping aimed to highlight the specific clinical relevance of posterior circulation impairment in dizziness and short-term outcomes. Carotid ultrasound recorded stenosis rate and plaque morphology; IMT was recorded as supporting data, not for grouping. Vertebral artery and BA stenosis were recorded separately. All images were preserved for review.

**Figure 1:**
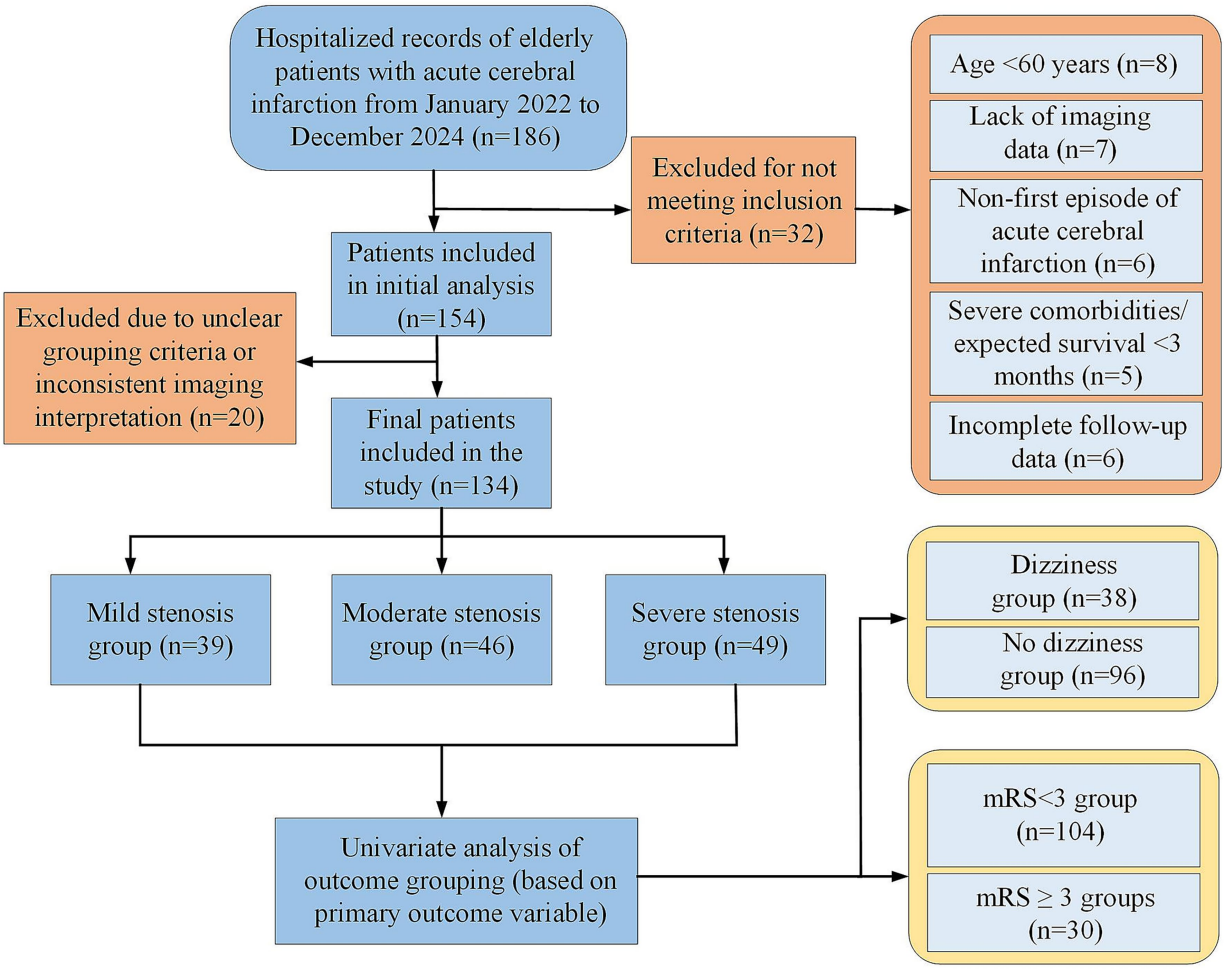
Flow chart of patient enrollment and exclusion.

To systematically analyze the relationship of variables with main outcomes (dizziness and poor neurological outcome [mRS ≥ 3] at 3 months), univariate analyses were conducted based on dizziness (dizziness group, *n* = 38; no-dizziness, *n* = 96) and mRS < 3 (*n* = 104) vs. mRS ≥ 3 (*n* = 30). Differences in clinical and imaging variables were compared (shown in Figure 1).

### 2.3 Infarction site determination

Head MRI or CT performed after admission was reviewed retrospectively. Two neurologists or neuroimaging physicians with attending-level or higher qualifications determined the main infarction sites based on imaging reports and source images. A senior physician reviewed cases when necessary. Infarction locations were classified according to vascular anatomy and perfusion territories: (1) anterior circulation cortical region (mainly frontal, parietal, temporal cortex); (2) anterior circulation subcortical region (basal ganglia, corona radiata); (3) cerebellar infarction; (4) brainstem infarction (pons, medulla, midbrain); (5) multifocal infarction (≥2 anatomical regions). Patients with cerebellar and/or brainstem lesions were categorized as having posterior circulation infarction (PCI) for subsequent analyses. All infarction classifications were based on existing clinical imaging and did not require additional examinations.

### 2.4 Clinical indicators and outcome measures

Baseline information included age, gender, common vascular risk factors (such as hypertension, diabetes, and atrial fibrillation), history of cardiovascular disease, smoking, previous stroke or transient ischemic attack, as well as medication history.

Dizziness assessment: all included patients were evaluated for dizziness symptoms at admission and at 3-month after discharge. The attending physician collected symptom details and performed neurological and balance examinations. Subjective dizziness severity was measured using the Dizziness Handicap Inventory (DHI) and visual analog scale (VAS) (12). Patients completed the scoring independently; family members assisted only when communication limitations existed, and the source of information was recorded. To document symptom changes during hospitalization, the most recent dizziness status and DHI/VAS scores within 24 h before discharge were recorded. These values were used only as clinical observations, not as formal analysis points. In this study, “dizziness” referred to subjective discomfort occurring after stroke onset and lasting at least 24 h, including one or more of the following: (1) vertigo: a spinning sensation involving self or surroundings; (2) imbalance/unsteadiness: instability while standing or walking, often with gait disturbance; (3) light-headedness: a sense of emptiness, floating, or faintness without clear spinning. Differentiation relied on detailed questioning, the Romberg test, cerebellar examinations, and balance testing. When patients could not clearly describe symptoms, caregivers could assist, and the information source was documented.

Neurological function assessment: All patients completed the National Institutes of Health Stroke Scale (NIHSS) (13) and modified Rankin Scale (mRS) scoring at admission and 3 months after discharge. The NIHSS obtained within 24 h before discharge served only as a clinical reference to describe short-term change and was not included in statistical analyses to avoid bias from variable hospital stays. Functional score data at 3 months were retrospectively collected through outpatient visits or phone calls, with all assessors uniformly trained to reduce subjective bias. An mRS score of 3 or above was defined as a poor neurological outcome. Information on whether the patient received acute-phase vascular recanalization therapy (intravenous thrombolysis or mechanical thrombectomy) was also recorded for later adjustment of confounding factors.

To minimize bias caused by different hospitalization lengths, discharge time was not used as an analysis node. NIHSS, dizziness status, and mRS recorded before discharge were not included in between-group comparisons or regression models. All statistical analyses were based on standardized time points: admission and 3 months after discharge. To ensure scoring consistency, all neurologists involved in NIHSS and mRS assessments underwent unified training and internal calibration before the study started. Data from 20 randomly selected patients at admission and 3 months were independently scored by two evaluators with associate chief physician qualifications or higher. Inter-rater agreement for NIHSS was analyzed using the intraclass correlation coefficient (ICC). Weighted kappa (*κ*) was used for mRS. All analyses used two-sided testing. Interpretation of ICC and weighted κ followed international standards (>0.75 indicating good agreement; >0.90 indicating excellent consistency).

### 2.5 Statistical analysis

All study data were double-entered independently by two investigators to ensure accuracy. Data collation and statistical analysis were performed using SPSS version 26.0. All continuous variables were tested for normality before analysis. Data with normal distribution were expressed as mean ± standard deviation. Group comparisons were performed using the t test or one-way analysis of variance (ANOVA). Non-normally distributed data were described as median with interquartile range, and the Mann–Whitney *U* test or Kruskal–Wallis *H* test was applied for between-group comparisons. Categorical variables were presented as counts and percentages. Group differences were assessed with the chi-square test or Fisher’s exact test.

To address the potential confounding effect of infarction location on dizziness, the proportions of the five infarction categories (anterior cortical, anterior subcortical, cerebellar, brainstem, and multifocal) and PCI were compared across the stenosis groups. The distribution of infarction sites was also compared between patients with dizziness and those without dizziness.

After univariate testing, variables with *p* < 0.10 were included in multivariable logistic regression models. Dizziness occurrence and a 3-month mRS ≥ 3 were used as dependent variables. Vascular stenosis classification served as the main independent variable. PCI, age, sex, and common risk factors were also entered into the models to examine the independent impact of stenosis severity and location on dizziness and adverse neurological outcomes. For continuous variables, multiple linear regression was used to evaluate their relationships with stenosis degree, DHI scores, and related factors. Missing retrospective evaluation data were handled using multiple imputation. All statistical tests were two-sided, with *p* < 0.05 considered statistically significant. All regression results are reported as regression coefficient, odds ratio (OR), 95% confidence interval (Cl), and *p-*value, according to statistical standards.

This study was retrospective. The sample size was determined by all consecutive patients who met the inclusion criteria between 2021 and 2024. Because no established effect size existed for the association between “vascular stenosis severity and dizziness or functional outcomes,” a prospective sample size calculation was not performed. Previous multivariable studies on intracranial or vertebrobasilar stenosis and stroke recurrence or poor outcomes showed that severe stenosis was usually associated with a two- to four-fold increase in risk (14, 15). Based on this expected range and assuming *α* = 0.05 and power = 0.80, approximately 120–150 patients were required for the main outcome analysis. The final sample of 134 cases met the general analytical requirement. However, some subgroups remained relatively small after stratification by stenosis severity, which may have limited statistical power. Further confirmation in larger cohorts is needed.

## 3 Results

### 3.1 Baseline characteristics

**Figure 2:**
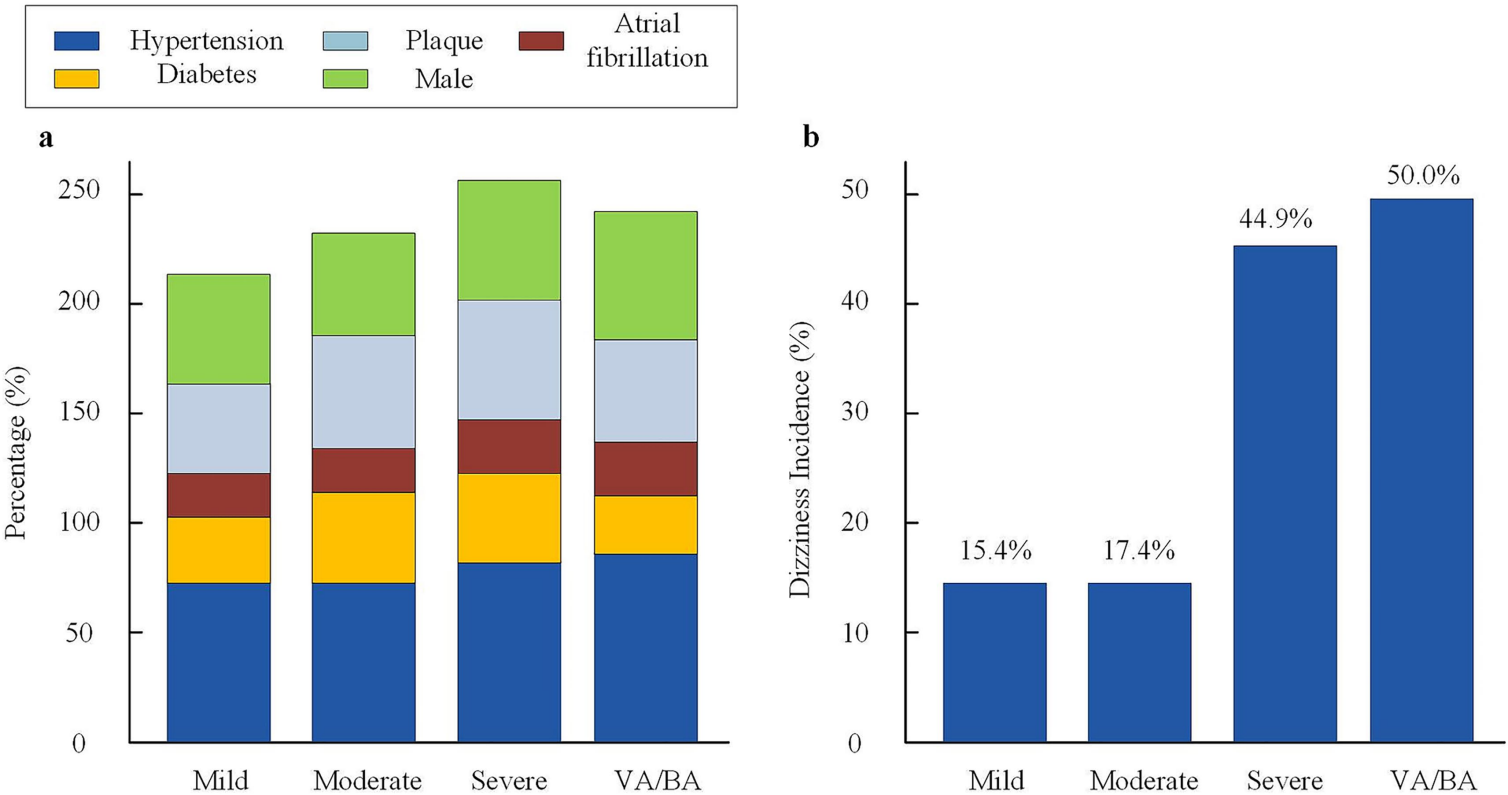
Baseline characteristics and dizziness incidence across stenosis groups. **(a)** Distribution of major vascular risk factors (hypertension, diabetes, plaque, atrial fibrillation, and male sex) among the four stenosis groups. **(b)** Incidence of dizziness in each stenosis group. Dizziness was defined as vertigo, imbalance, or light-headedness lasting ≥24 h. VA, vertebral artery; BA, basilar artery.

There was no statistical difference among groups in the proportions of age, gender, hypertension, diabetes, atrial fibrillation, or plaque. The severe stenosis group had 75.5% with hypertension, 57.1% with plaque, and 57.1% males; in the mild group these were 71.8, 43.6, and 53.8%, respectively. The moderate group had similar distributions. In the VA/BA stenosis group, the proportion with hypertension was 78.6%, plaque was 52.4%, and males were 59.5%. The differences in risk factor composition and disease burden among the groups were not statistically significant (*p* > 0.05). This indicates comparability among groups and helps reduce confounding for outcome analysis (shown in Figure 2 and Table 1).

**Table 1:** Baseline characteristics of patients in different stenosis groups (*n* = 134).

Variable	Mild (*n* = 39)	Moderate (*n* = 46)	Severe (*n* = 49)	VA/BA (*n* = 42)	*F*/*χ*^2^/Fisher	*p*
Age (years, mean ± SD)	70.9 ± 6.3	71.5 ± 7.2	72.8 ± 7.0	72.6 ± 7.1	1.108	0.333
Male [*n*, (%)]	21 (53.8%)	24 (52.2%)	28 (57.1%)	25 (59.5%)	0.305	0.858
Hypertension [*n*, (%)]	28 (71.8%)	33 (71.7%)	37 (75.5%)	33 (78.6%)	0.245	0.885
Diabetes [*n*, (%)]	12 (30.8%)	17 (37.0%)	19 (38.8%)	14 (33.3%)	0.567	0.753
Atrial fibrillation [*n*, (%)]	7 (17.9%)	9 (19.6%)	12 (24.5%)	11 (26.2%)	0.666	0.717
Coronary heart disease [*n*, (%)]	9 (23.1%)	13 (28.3%)	13 (26.5%)	11 (26.2%)	0.320	0.852
History of smoking [*n*, (%)]	14 (35.9%)	15 (32.6%)	17 (34.7%)	15 (35.7%)	0.139	0.934
History of cerebral infarction [*n*, (%)]	6 (15.4%)	8 (17.4%)	10 (20.4%)	10 (23.8%)	0.390	0.823
NIHSS score at admission (mean ± SD)	3.4 ± 2.2	4.1 ± 2.7	4.7 ± 2.6	4.8 ± 2.7	2.013	0.138
mRS score at admission (mean ± SD)	1.8 ± 1.0	2.0 ± 1.1	2.2 ± 1.1	2.2 ± 1.2	1.512	0.223
Carotid artery plaque [*n*, (%)]	17 (43.6%)	23 (50.0%)	28 (57.1%)	22 (52.4%)	1.392	0.499
Intima-media thickness (mm, mean ± SD)	1.04 ± 0.19	1.10 ± 0.21	1.14 ± 0.22	1.13 ± 0.21	1.970	0.143
Vertebral/BA stenosis [*n*, (%)]	6 (15.4%)	13 (28.3%)	23 (46.9%)	1.13 ± 0.21	11.663	0.003
Acute phase recanalization therapy [*n*, (%)]	5 (12.8%)	7 (15.2%)	9 (18.4%)	7 (16.7%)	0.533	0.766
Infarction location	–	–	–	–	–	–
Anterior circulation cortex	18 (46.2%)	21 (45.7%)	20 (40.8%)	5 (16.7%)	8.72	0.013
Anterior circulation subcortex	14 (35.9%)	15 (32.6%)	16 (32.7%)	4 (13.3%)	8.27	0.041
Cerebellum	3 (7.7%)	4 (8.7%)	7 (14.3%)	14 (46.7%)	Fisher	<0.001
Brainstem	1 (2.6%)	2 (4.3%)	3 (6.1%)	10 (33.3%)	Fisher	<0.001
Multifocal infarction	3 (7.7%)	4 (8.7%)	3 (6.1%)	7 (23.3%)	8.48	0.037
PCI	4 (10.3%)	6 (13.0%)	10 (20.4%)	25 (83.3%)	54.91	<0.001
TOAST subtype	–	–	–	–	–	–
Large-artery atherosclerosis	19 (48.7%)	24 (52.2%)	27 (55.1%)	20 (47.6%)	1.12	0.772
Cardioembolism	6 (15.4%)	7 (15.2%)	8 (16.3%)	7 (16.7%)	0.05	0.996
Small-vessel occlusion	7 (17.9%)	8 (17.4%)	6 (12.2%)	6 (14.3%)	0.81	0.848
Other	7 (17.9%)	7 (15.2%)	8 (16.3%)	9 (21.4%)	0.84	0.839

VA/BA, vertebral/basilar artery; VB, vertebrobasilar; PCI, posterior circulation ischemia; mRS, modified Rankin Scale; NIHSS, National Institutes of Health Stroke Scale; TOAST, Trial of ORG 10172 in Acute Stroke Treatment.

There were significant differences in the distribution of infarction sites among the vascular stenosis groups. Cerebellar and brainstem infarctions were markedly more frequent in the VA/BA group (both *p* < 0.001). Differences were also observed in anterior cortical, anterior subcortical, and multifocal infarctions across the four groups (*p* = 0.013, 0.041, and 0.037). PCI had the highest proportion in the VA/BA group (83.3%), with a highly significant difference (*p* < 0.001). These findings indicated that infarction location might act as a confounding factor influencing dizziness, and thus it was included in the multivariable adjustments. In addition, the distribution of TOAST was comparable among the four stenosis groups, with no significant differences (*p* > 0.05) (Table 1).

### 3.2 Relationship between degree of vascular stenosis and dizziness symptoms

The incidence and clinical features of dizziness were compared among elderly patients with acute ischemic stroke grouped by degree of vascular stenosis. Results showed that the incidence of dizziness in the severe stenosis group was 44.9% (22/49), which was significantly higher than in the mild group (15.4%, 6/39) and the moderate group (17.4%, 8/46), with statistical significance (*χ*^2^ = 11.23, *p* = 0.004). The incidence of dizziness in the VA/BA stenosis group was 50.0% (21/42), also higher than in patients with other types of stenosis (*p* = 0.001). The severe stenosis group had a DHI score of 35.7 ± 11.4 and a VAS score of 6.1 ± 1.9. The VA/BA stenosis group had a DHI score of 37.9 ± 12.2 and a VAS score of 6.1 ± 1.9. Both were significantly higher than those in the mild and moderate groups (DHI 20.8 ± 7.5 and 23.1 ± 8.2, VAS 3.0 ± 1.2 and 3.4 ± 1.5; all *F* values >19, *p* < 0.001). In addition, the proportions of persistent dizziness and accompanying balance disorder in the severe group and VA/BA stenosis group were higher than in other groups (*p* < 0.01). The data suggest that the higher the degree of stenosis, especially with vertebral or BA involvement, the higher the incidence and severity of dizziness (see Supplementary Table 2).

### 3.3 Relationship between degree of vascular stenosis and neurological function scores

To further investigate the impact of vascular stenosis on neurological deficits and prognosis, NIHSS scores at admission and retrospective mRS evaluation results within 3 months were analyzed among the groups. The NIHSS scores at admission for the severe stenosis group and VA/BA stenosis group were 5.2 ± 2.4 and 5.3 ± 2.3, both higher than those in the mild group (3.1 ± 1.7) and moderate group (3.6 ± 2.0), with statistical significance (*F* = 9.72, *p* < 0.001). At 3 months, the mRS scores in the severe group and VA/BA group were 2.6 ± 1.2 and 2.8 ± 1.3, also higher than the mild group (1.7 ± 0.9) and moderate group (2.0 ± 1.1), with significant differences (*F* = 8.61, p < 0.001). The proportions of patients with mRS ≥ 3 (indicating dependence or worse) at 3 months were 38.8% in the severe group and 42.9% in the VA/BA group, higher than in the mild group (10.3%) and moderate group (15.2%) (*χ*^2^ = 13.85, *p* = 0.001). The 3-month mortality rate in the mild stenosis group was 0, 2.2% in the moderate group, 8.2% in the severe group, and 9.5% in the VA/BA group, with no statistical significance among groups (*p* = 0.073). Multivariate logistic regression analysis showed that severe VA/BA stenosis was an independent risk factor for poor neurological outcome (mRS ≥ 3 at 3 months) (OR = 2.91, 95%CI: 1.32–6.41, *p* = 0.008) (see Supplementary Table 3).

**Figure 3:**
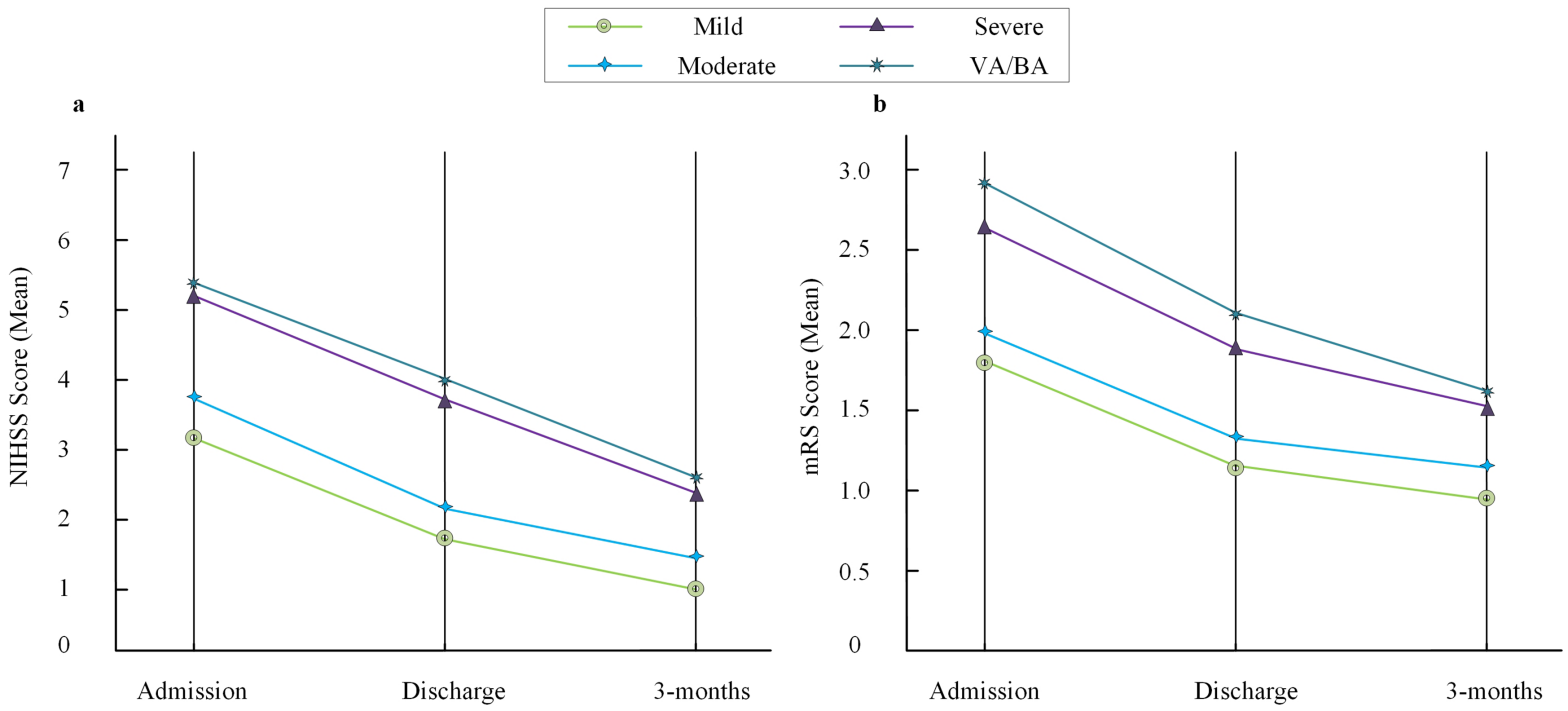
Trends of NIHSS and mRS scores across stenosis groups. **(a)** Mean NIHSS scores at admission, discharge, and 3-month follow-up in the four stenosis groups (mild, moderate, severe, and VA/BA). **(b)** Mean mRS scores at the same three time points. The discharge time point is included for descriptive trend visualization only and was not used for statistical inference due to individual variability in discharge timing. NIHSS, National Institutes of Health Stroke Scale; mRS, modified Rankin Scale; VA, vertebral artery; BA, basilar artery.

The trends of NIHSS and mRS scores at different time points were further displayed by group. It was found that the severe stenosis group and the VA/BA group had higher NIHSS and mRS scores than the mild and moderate groups at admission, discharge, retrospective assessment points within 3 months, and although the scores in all groups decreased over time, the differences among groups persisted (shown in Figure 3).

### 3.4 Univariate analysis

To investigate the relationship between clinical and imaging variables and poor neurological outcomes at 3 months, a univariate analysis of major baseline variables was conducted. The results showed that the mean age in the poor prognosis group (mRS ≥ 3 at 3 months) was 74.5 ± 7.4 years, significantly higher than the good prognosis group (71.1 ± 6.5 years, *t* = 2.33, *p* = 0.021). The poor outcome group had a higher admission NIHSS score (5.7 ± 2.6) compared to the good prognosis group (3.5 ± 1.8, *t* = 4.77, *p* < 0.001). Among patients with poor outcomes, the proportion of severe stenosis was 90.0%, much higher than in the good prognosis group (21.2%, *χ*^2^ = 45.93, *p* < 0.001). The rate of VA/BA stenosis in the poor outcome group was 73.3%, also much higher than the good prognosis group (19.2%, *χ*^2^ = 29.77, *p* < 0.001). Diabetes and atrial fibrillation were more common in the poor outcome group, but the differences were not statistically significant. Gender, hypertension, and acute recanalization treatment did not show obvious correlation with poor outcome. These results suggest that severe vascular stenosis, VA/BA stenosis, older age, and higher admission NIHSS score are important correlates of poor 3-month prognosis in elderly patients with acute ischemic stroke (see Table 2).

**Table 2:** Univariate analysis between mRS < 3 and mRS ≥ 3 groups at 3 months (*n* = 134).

Variable	mRS < 3 group (*n* = 104)	mRS ≥ 3 group (*n* = 30)	Statistic	*p*-value
Age (years, mean ± SD)	71.1 ± 6.5	74.5 ± 7.4	*t* = 2.33	0.021
Male [*n*, %]	54 (51.9%)	19 (63.3%)	*χ*^2^ = 1.29	0.256
Hypertension [*n*, %]	74 (71.2%)	24 (80.0%)	*χ*^2^ = 0.94	0.332
Diabetes [*n*, %]	28 (26.9%)	10 (33.3%)	*χ*^2^ = 0.46	0.498
Atrial fibrillation [*n*, %]	15 (14.4%)	8 (26.7%)	*χ*^2^ = 2.32	0.128
NIHSS score (mean ± SD)	3.5 ± 1.8	5.7 ± 2.6	*t* = 4.77	<0.001
Mild stenosis [*n*, %]	38 (36.5%)	1 (3.3%)	*χ*^2^ = 13.42	<0.001
Moderate stenosis [*n*, %]	44 (42.3%)	2 (6.7%)	*χ*^2^ = 15.72	<0.001
Severe stenosis [*n*, %]	22 (21.2%)	27 (90.0%)	*χ*^2^ = 45.93	<0.001
VA/BA stenosis [*n*, %]	20 (19.2%)	22 (73.3%)	*χ*^2^ = 29.77	<0.001
Acute recanalization [*n*, %]	15 (14.4%)	6 (20.0%)	*χ*^2^ = 0.54	0.464

VA/BA, vertebral/basilar artery; mRS, modified Rankin Scale; NIHSS, National Institutes of Health Stroke Scale.

To further clarify the associations between clinical/imaging variables and dizziness, patients were divided by the presence or absence of dizziness for univariate analysis. The results indicated that the proportion of severe stenosis in the dizziness group (57.9%) was much higher than that in the non-dizziness group (28.1%, *χ*^2^ = 10.29, *p* = 0.001). The rate of VA/BA stenosis was also higher in the dizziness group (55.3% vs. 21.9%, *χ*^2^ = 13.51, *p* < 0.001). Furthermore, the admission NIHSS score was higher in the dizziness group (4.6 ± 2.3 vs. 3.8 ± 2.1, *t* = 2.00, *p* = 0.047). No significant differences were found between groups in age, sex, hypertension, diabetes, atrial fibrillation, carotid plaque, intima-media thickness, or acute recanalization (all *p* > 0.05). These findings suggest that the severity of vascular stenosis and VA/BA stenosis are important factors related to dizziness, and NIHSS score is also associated with dizziness. In addition, The proportion of PCI was significantly higher in the dizziness group than in the non-dizziness group (65.8% vs. 19.8%, *χ*^2^ = 24.31, *p* < 0.001). The rates of cerebellar infarction and brainstem infarction were also higher in the dizziness group, at 47.4% vs. 10.4% (p < 0.001) and 23.7% vs. 7.3% (*p* = 0.008), respectively. In contrast, anterior circulation cortical infarction was more common in the non-dizziness group (55.2% vs. 28.9%, *p* = 0.005) (see Table 3).

**Table 3:** Univariate analysis of clinical and imaging variables between dizziness and non-dizziness groups (*n* = 134).

Variable	Dizziness group (*n* = 38)	Non-dizziness group (*n* = 96)	Statistic	*p*-value
Age (years, mean ± SD)	72.1 ± 7.0	71.8 ± 6.8	*t* = 0.22	0.827
Male [*n*, %]	22 (57.9%)	49 (51.0%)	*χ*^2^ = 0.51	0.476
Hypertension [*n*, %]	29 (76.3%)	66 (68.8%)	*χ*^2^ = 0.71	0.400
Diabetes [*n*, %]	13 (34.2%)	25 (26.0%)	*χ*^2^ = 0.92	0.338
Atrial fibrillation [*n*, %]	9 (23.7%)	14 (14.6%)	*χ*^2^ = 1.66	0.198
NIHSS score (mean ± SD)	4.6 ± 2.3	3.8 ± 2.1	*t* = 2.00	0.047
Severe stenosis [*n*, %]	22 (57.9%)	27 (28.1%)	*χ*^2^ = 10.29	0.001
VA/BA stenosis [*n*, %]	21 (55.3%)	21 (21.9%)	*χ*^2^ = 13.51	<0.001
Carotid plaque [*n*, %]	21 (55.3%)	47 (49.0%)	*χ*^2^ = 0.38	0.538
Intima-media thickness (mm, mean ± SD)	1.11 ± 0.20	1.08 ± 0.21	*t* = 0.81	0.419
Acute recanalization [*n*, %]	8 (21.1%)	13 (13.5%)	*χ*^2^ = 1.24	0.265
Cerebellar infarction [*n*, %]	18 (47.4%)	10 (10.4%)	*χ*^2^ = 17.65	<0.001
Brainstem infarction [*n*, %]	9 (23.7%)	7 (7.3%)	*χ*^2^ = 6.92	0.008
Anterior circulation cortical infarction [*n*, %]	11 (28.9%)	53 (55.2%)	*χ*^2^ = 8.03	0.005
PCI total [*n*, %]	25 (65.8%)	19 (19.8%)	*χ*^2^ = 24.31	<0.001

VA/BA, vertebral/basilar artery; NIHSS, National Institutes of Health Stroke Scale; PCI, posterior circulation ischemia.

### 3.5 Inter-rater consistency

To evaluate the stability and reliability of neurological scale assessments, two independent raters scored 20 randomly selected patients. The results showed excellent inter-rater agreement for the NIHSS, with an ICC of 0.92 (95% CI: 0.88–0.97). The agreement for the mRS was also high, with a weighted kappa coefficient of 0.87 (95% CI: 0.79–0.95). These findings indicated good consistency between raters for both NIHSS and mRS, supporting the reliability of the scoring data for subsequent statistical analyses.

### 3.6 Multivariate analysis

**Figure 4:**
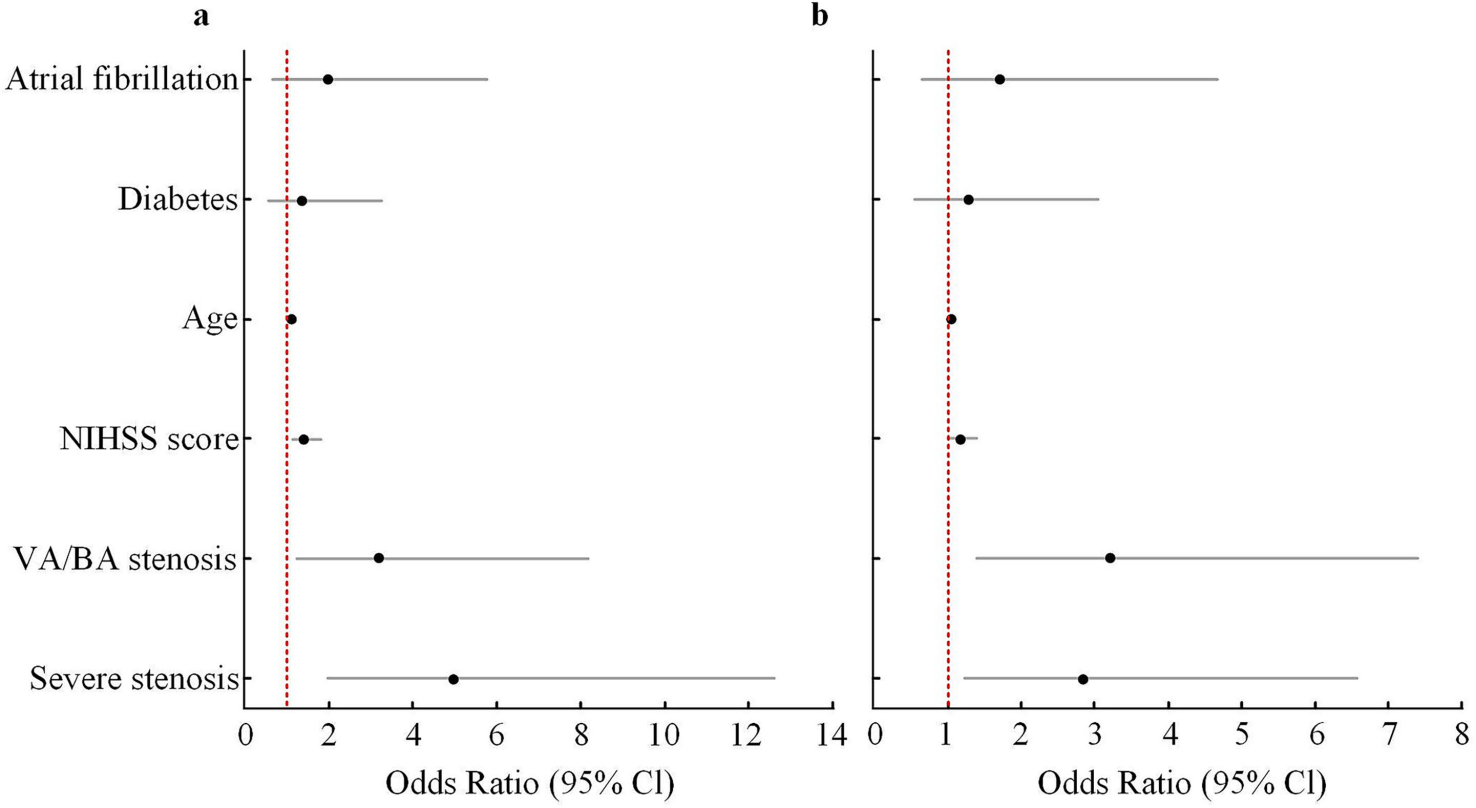
Multivariate logistic regression analysis of independent predictors for poor outcome (mRS ≥ 3) and dizziness at 3 months. **(a)** Forest plot showing adjusted ORs and 95% CIs for predictors of poor functional outcome (mRS ≥ 3) at 3 months. **(b)** Forest plot showing adjusted ORs and 95% CIs for predictors of dizziness at 3 months. Variables included in the multivariate models were age, diabetes, atrial fibrillation, baseline NIHSS score, severe stenosis, and VA/BA stenosis. Severe stenosis and VA/BA stenosis remained independent predictors in the adjusted models. The red dashed line represents an OR of 1.0. OR, odds ratio; CI, confidence interval; NIHSS, National Institutes of Health Stroke Scale; VA, vertebral artery; BA, basilar artery.

In the multivariable logistic regression model using dizziness as the outcome, and after adjustment for age, diabetes, atrial fibrillation, and admission NIHSS score, VA/BA stenosis remained significantly associated with dizziness (OR = 3.42, 95% CI: 1.28–9.13, *p* = 0.014). PCI was also a strong correlate of dizziness (OR = 4.51, 95% CI: 2.01–10.13, *p* < 0.001). The OR for severe stenosis was 1.86 (95% CI: 0.88–3.93, *p* = 0.102), which did not reach statistical significance. In the model using 3-month mRS ≥ 3 as the outcome, severe vascular stenosis (OR = 4.96, 95% CI: 1.95–12.62, *p* = 0.001), VA/BA stenosis (OR = 3.18, 95% CI: 1.23–8.20, *p* = 0.017), and admission NIHSS score (OR = 1.42, 95% CI: 1.13–1.78, *p* = 0.002) were all independent predictors of poor functional outcome. PCI did not enter this model. Age had a mild effect on prognosis (OR = 1.10, *p* = 0.036). Diabetes and atrial fibrillation were not significant in either model (shown in Figure 4 and Table 4).

**Table 4:** Independent factors for main outcomes by multivariate logistic regression.

Variable	Dizziness (OR, 95% CI)	*p*-value	3-month mRS ≥ 3 (OR, 95% CI)	*p*-value
Severe stenosis	1.86 (0.88–3.93)	0.102	4.96 (1.95–12.62)	0.001
VA/BA stenosis	3.42 (1.28–9.13)	0.014	3.18 (1.23–8.20)	0.017
PCI	4.51 (2.01–10.13)	<0.001	–	–
NIHSS score	1.18 (0.99–1.42)	0.061	1.42 (1.13–1.78)	0.002
Age	1.02 (0.98–1.06)	0.311	1.10 (1.01–1.21)	0.036
Diabetes	1.29 (0.54–3.06)	0.566	1.35 (0.55–3.30)	0.517
Atrial fibrillation	1.72 (0.63–4.70)	0.286	1.98 (0.68–5.77)	0.209

VA/BA, vertebral/basilar artery; mRS, modified Rankin Scale; NIHSS, National Institutes of Health Stroke Scale.

### 3.7 Survival analysis and functional outcomes

**Figure 5:**
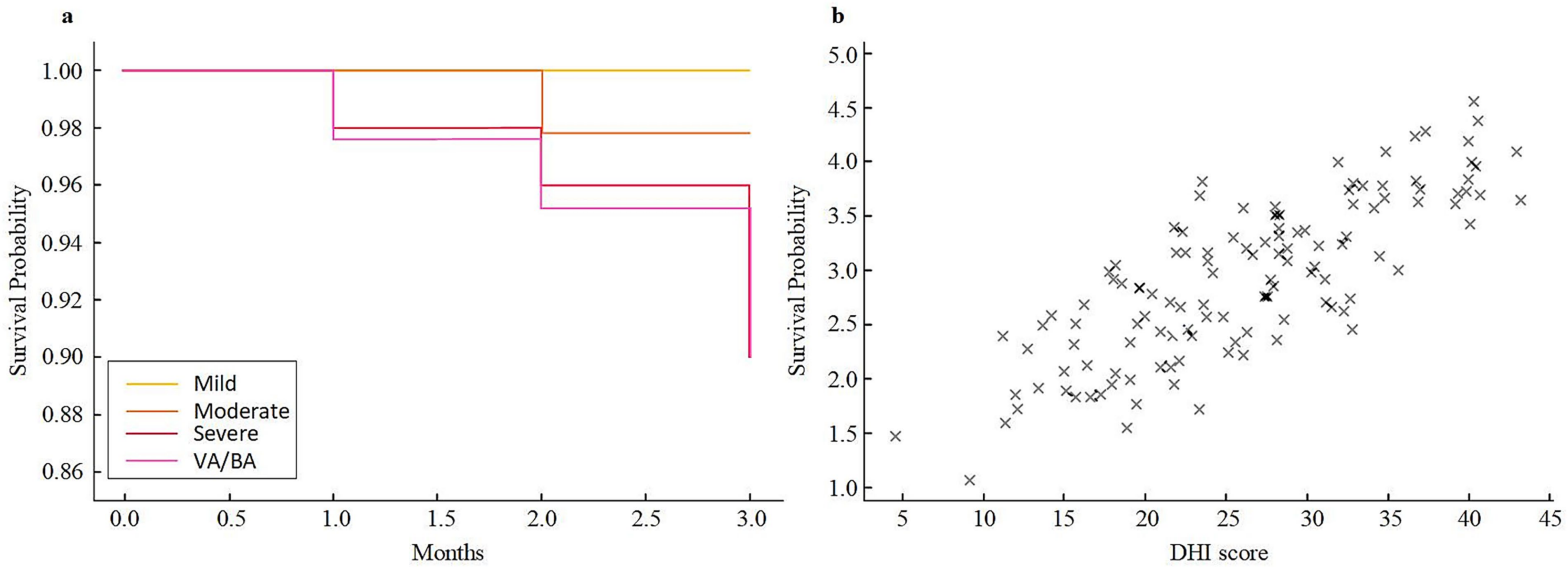
Three-month survival and correlation analysis in different stenosis groups. **(a)** Kaplan–Meier survival curves for patients with mild, moderate, severe, and VA/BA stenosis. **(b)** Scatter plot illustrating the relationship between DHI scores and mRS scores at 3 months. VA/BA, vertebral/basilar artery; DHI, Dizziness Handicap Inventory; mRS, modified Rankin Scale.

The Kaplan–Meier survival curve showed that patients in the severe stenosis and VA/BA stenosis groups had a lower survival probability within 3 months than those in the mild and moderate groups. At 3 months, the survival rates were 100.0 and 97.8% in the mild and moderate groups, respectively, while they were 92.0 and 90.0% in the severe and VA/BA groups. Correlation analysis showed a positive relationship between DHI score and mRS score at 3 months (*r* = 0.48, *p* < 0.001). Higher DHI scores were linked to higher mRS scores (shown in Figure 5).

## 4 Discussion

### 4.1 Summary of the main findings

This study focused on elderly patients with acute ischemic stroke and systematically analyzed the effects of the degree and location of vascular stenosis on dizziness and short-term neurological outcomes (16). The results demonstrated that VA/BA stenosis not only increased the risk of dizziness but were also closely associated with a higher risk of functional dependence (mRS ≥ 3) at 3 months. Higher NIHSS scores at admission and older age were also found to be independent predictors of poor outcomes.

### 4.2 Comparison with previous studies

Compared to previous reports, this study further detailed the quantitative analysis of stenosis grouping and clinical outcomes, and specifically discussed the independent effect of VA/BA stenosis and its contribution to dizziness (17, 18). Most published articles have focused on endpoints such as stroke recurrence and death, while this research used DHI and mRS scores to provide a more comprehensive assessment of dizziness and its impact on daily functioning (19). The current findings are consistent with international stroke cohort studies that conclude the degree of stenosis determines disability risk (20), but this work especially highlights the association between posterior circulation stenosis and dizziness in elderly Chinese patients, which is a new insight.

Elderly patients often present with overlapping dizziness-related symptoms, including vertigo, imbalance, and light-headedness (21). Their boundaries are not completely clear in clinical settings. Based on this characteristic, the three symptom types were differentiated and documented during assessment, but they were classified together as “dizziness” for statistical analysis. This approach improved interpretability and clinical utility. It was also consistent with how dizziness has been treated in previous stroke studies and reflected symptom patterns commonly seen in older adults.

### 4.3 Summary of key neurological evidence

To further clarify the neurological basis supporting the associations found in this study, Supplementary Table 7 (14, 22–25) was included to summarize representative evidence related to vascular stenosis, dizziness, and functional outcomes. Existing literature consistently indicates that vertebrobasilar stenosis is a major cause of posterior circulation ischemia and is closely linked to higher risks of short-term recurrence and disability. Dizziness and vertigo have strong localizing value in posterior circulation hypoperfusion and often indicate cerebellar or brainstem involvement. Heavier stenosis burden is also associated with poorer neurological recovery after stroke. These findings aligned well with the results of this study, including the higher dizziness rate in VA/BA stenosis and the association between severe stenosis and functional dependence at 3 months. The neurological evidence thus provided a solid foundation for interpreting the study results. Several reviews have emphasized that dizziness in elderly patients should not be considered a benign symptom. Instead, vascular imaging should be performed to evaluate the possibility of posterior circulation insufficiency. This view fits with the design and logic of this study, which combined stenosis severity, infarction site, and dizziness characteristics to better identify high-risk individuals.

In addition, the study analyzed the distribution of TOAST (Table 1). The TOAST subtypes were evenly distributed across stenosis groups. As a mechanism-based classification, TOAST represents a different pathological dimension and was unlikely to confound the main associations between stenosis, dizziness, and clinical outcomes.

### 4.4 Mechanisms linking posterior circulation stenosis, dizziness, and infarction sites

Dizziness and vertigo are typical localizing symptoms of ischemia in the vertebrobasilar system and often indicate involvement of the cerebellum or brainstem, which contain key vestibular structures. Prior studies have shown that dizziness may have prognostic implications in posterior circulation stroke (26). In this study, the markedly higher dizziness rate in the VA/BA stenosis group supported this localizing and prognostic value. Severe stenosis was also associated with a higher dizziness rate (44.9%) than mild or moderate stenosis, and VA/BA stenosis showed an even higher rate (50.0%). These results were consistent with multicenter studies such as the WASID trial, which reported a link between stenosis severity and vestibular symptoms (27, 28).

Neuroimaging and clinical studies indicate that many central ischemic vertigo syndromes originate from regions adjacent to the midline or lateral medulla/pons. These areas are key components of vestibular pathways and balance control (29). When posterior circulation flow decreases, dizziness and imbalance are more likely to occur. The main mechanism is that the vertebrobasilar system supplies the brainstem, cerebellum, and vestibular pathways. Reduced perfusion can impair vestibular function and lead to spinning sensations, imbalance, and persistent dizziness. Consistent with this mechanism, cerebellar and brainstem infarctions were significantly more frequent in the dizziness group, indicating that dizziness reflected both hypoperfusion and the topographic location of infarction.

Another mechanism described in previous literature is that isolated dizziness may arise from ischemia in the Anterior Inferior Cerebellar Artery (AICA) territory or small insular cortex infarctions (30). However, based on the infarction site comparisons in this study, no isolated AICA infarction or isolated insular infarction was found. Instead, infarction patterns in the dizziness group mainly involved cerebellar and brainstem regions supplied by the vertebrobasilar system. Therefore, these alternative mechanisms did not apply to this cohort and were unlikely to affect the main associations observed.

### 4.5 Association between vascular stenosis and poor functional outcomes

During the 3-month retrospective evaluation, the proportions of patients with functional dependence (mRS ≥ 3) in the severe and VA/BA groups were 38.8 and 42.9%, respectively. Severe stenosis causes local brain tissue to remain in a state of persistent low perfusion or even ischemia, which not only impairs the repair and reconstruction of neural networks but also increases the chance of recurrent stroke and complications. Large prospective studies abroad, such as the SPARCL study, have pointed out that vascular stenosis is a key determinant of post-stroke disability, and the current data on elderly patients further enrich this conclusion (31). The negative impact of VA/BA stenosis on outcomes is also significant, possibly due to the slow recovery and high disability risk after damage to the posterior circulation supply region, which is consistent with international recommendations for vigilance regarding posterior circulation stroke (32).

### 4.6 Relationship between dizziness severity and functional outcomes

Correlation analysis showed a moderate positive relationship between DHI score and mRS score at 3 months, suggesting that more severe subjective dizziness is linked to worse objective functional impairment. Dizziness not only reflects vestibular dysfunction but also usually signals decreased ability in daily activities and reduced compliance with rehabilitation. Similar findings have been reported in many previous clinical studies. For example, a Japanese prospective cohort found that dizziness scales such as DHI can help predict the speed of functional recovery after stroke. The results here support the value of including dizziness in outcome assessment (33).

### 4.7 Effects of NIHSS and age on prognosis

Further regression analysis revealed that for every one-point increase in admission NIHSS score, the risk of mRS ≥ 3 at 3 months rose (OR = 1.42). Similarly, each additional year of age was associated with about a 10% increase in functional dependence risk. The NIHSS score accurately reflects the severity of neurological injury and multisystem involvement during the acute phase of stroke, and patients with higher scores generally have less potential for recovery. This is in line with long-term follow-up results from international stroke registries (34). Age is an important factor for poor prognosis because it affects both neural repair and plasticity, as well as being related to accumulated chronic diseases and more complications. Many European and American stroke studies have included age in their risk models, and these results further support this perspective (32).

### 4.8 Clinical implications

The study indicated that vascular imaging has important value in risk stratification and management of elderly stroke patients. Those with severe stenosis or VA/BA stenosis may require closer monitoring, earlier intervention, and individualized secondary prevention strategies. Dizziness should also be recognized and managed because it affects quality of life and may increase the risk of functional decline. These findings highlight the key role of stenosis severity, stenosis location, and dizziness symptoms in clinical decision-making for elderly patients with acute ischemic stroke.

### 4.9 Study limitations

This study had several limitations. First, although the total sample size was sufficient for the main analyses, subgroup sample sizes were limited after stratifying stenosis severity. Event numbers for dizziness and poor outcomes (mRS ≥ 3) were also relatively small, which may have widened confidence intervals and reduced statistical power. Second, the retrospective single-center design and incomplete follow-up may have underestimated adverse events. Third, some potential confounders, such as cardioembolic stroke mechanisms or treatment differences, were not fully controlled. Fourth, long-term outcomes were not included, preventing the evaluation of extended prognosis. In addition, white-matter changes and other markers of small-vessel disease were not assessed and may have had effects on dizziness symptoms (35).

### 4.10 Future directions

Future studies may expand in several areas. First, combining TOAST subtypes and markers of small-vessel disease, such as white-matter hyperintensity, may allow a more systematic stratified analysis. Second, large multicenter prospective studies could better clarify causal pathways among stenosis, dizziness, and outcomes. Third, including long-term follow-up and quality-of-life assessments may help evaluate the full impact of dizziness on stroke recovery.

## 5 Conclusion

In conclusion, the study indicates that VA/BA stenosis and PCI are the main independent factors associated with dizziness in elderly patients with acute ischemic stroke. Severe vascular stenosis and VA/BA stenosis markedly increase the risk of functional dependence at 3 months (mRS ≥ 3). Higher NIHSS scores at admission and advanced age are also independent predictors of poor outcomes. Clinical practice should emphasize comprehensive vascular imaging assessment of the head and neck, with particular attention to high-risk individuals presenting with severe stenosis or vertebrobasilar involvement. Early risk management and symptom-focused interventions may help improve short-term functional recovery and quality of life for stroke patients.

## Data Availability

The raw data supporting the conclusions of this article will be made available by the authors, without undue reservation.
