# Supplementary material for: Correlation between vascular stenosis severity and dizziness symptoms and neurological prognosis in elderly patients with acute ischemic stroke
Source: Front Neurol. 2026 Jan 12;16:1730875. doi: 10.3389/fneur.2025.1730875 (PMC12833226; doi:10.3389/fneur.2025.1730875)
Supplement: Supplementary file 1 [file Data_Sheet_1.docx]

Supplementary Material

# Supplementary Tables

**Supplementary Table 1. Dizziness manifestations and severity in patients with different vascular stenosis groups (n=134)**

| Indicator | Mild (n=39) | Moderate (n=46) | Severe (n=49) | VA/BA (n=42) | F/χ² | P |
| --- | --- | --- | --- | --- | --- | --- |
| Incidence of dizziness [n, %] | 6 (15.4%) | 8 (17.4%) | 22 (44.9%) | 21 (50.0%) | 11.23 | 0.004 |
| Persistent dizziness [n, %] | 2 (5.1%) | 3 (6.5%) | 15 (30.6%) | 14 (33.3%) | 15.67 | <0.001 |
| Rotational sensation [n, %] | 2 (5.1%) | 3 (6.5%) | 12 (24.5%) | 12 (28.6%) | 10.22 | 0.006 |
| Balance disorder [n, %] | 1 (2.6%) | 2 (4.3%) | 8 (16.3%) | 8 (19.0%) | 8.95 | 0.030 |
| Nausea and vomiting [n, %] | 1 (2.6%) | 2 (4.3%) | 7 (14.3%) | 7 (16.7%) | 7.45 | 0.040 |
| Falls or unsteadiness [n, %] | 0 (0.0%) | 1 (2.2%) | 5 (10.2%) | 5 (11.9%) | 6.83 | 0.055 |
| DHI score (Mean ± SD) | 20.8 ± 7.5 | 23.1 ± 8.2 | 35.7 ± 11.4 | 37.9 ± 12.2 | 23.47 | <0.001 |
| VAS score (Mean ± SD) | 3.0 ± 1.2 | 3.4 ± 1.5 | 6.1 ± 1.9 | 6.6 ± 2.1 | 19.28 | <0.001 |
| Dizziness at discharge [n, %] | 1 (2.6%) | 2 (4.3%) | 12 (24.5%) | 11 (26.2%) | 13.13 | 0.002 |

Note: “Dizziness at discharge” was treated as a short-term clinical observation during hospitalization. It was used only for descriptive statistics and was not included as a study endpoint or a variable in multivariable analyses, in order to avoid bias caused by differences in hospital stay length. Abbreviations: VA/BA = vertebral/basilar artery; DHI = Dizziness Handicap Inventory; VAS = visual analog scale

**Supplementary Table 2. Neurological function scores and prognosis in patients with different vascular stenosis groups (n=134)**

| Indicator | Mild (n=39) | Moderate (n=46) | Severe (n=49) | VA/BA (n=42) | F/χ² | P |
| --- | --- | --- | --- | --- | --- | --- |
| NIHSS score at admission (Mean±SD) | 3.1 ± 1.7 | 3.6 ± 2.0 | 5.2 ± 2.4 | 5.3 ± 2.3 | 9.72 | <0.001 |
| mRS score at 3 months (Mean±SD) | 1.7 ± 0.9 | 2.0 ± 1.1 | 2.6 ± 1.2 | 2.8 ± 1.3 | 8.61 | <0.001 |
| mRS≥3 at 3 months [n, %] | 4 (10.3%) | 7 (15.2%) | 19 (38.8%) | 18 (42.9%) | 13.85 | 0.001 |
| Death at 3 months [n, %] | 0 (0.0%) | 1 (2.2%) | 4 (8.2%) | 4 (9.5%) | 5.22 | 0.073 |
| Multivariate regression: VA/BA severe stenosis vs others | - | - | OR=2.91 (1.32–6.41) | - | - | 0.008 |

Note: Abbreviations: VA/BA = vertebral/basilar artery; National Institutes of Health Stroke Scale = NIHSS; mRS = modified Rankin Scale.

**Supplementary Table 3. Key Evidence Supporting the Relationship Between Vascular Stenosis, Dizziness, and Functional Outcomes**

| Study | Key Finding | Direct Relevance to This Study |
| --- | --- | --- |
| Abuzinadah et al., 2016 | Symptomatic VB stenosis markedly increases the risk of recurrent stroke and death. | Supports the high-risk nature of VB stenosis and justifies analyzing it as an independent vascular factor. |
| Gulli et al., 2009 | VB stenosis is strongly linked to high early recurrence after posterior circulation stroke or TIA. | Reinforces the importance of VB involvement in early neurological deterioration. |
| Li et al., 2021 | Severe VB stenosis is associated with increased rates of recurrent ischemic events and disability. | Provides evidence that severe stenosis relates to worse functional outcomes, aligning with the mRS findings of this study. |
| Halmágyi et al., 2023 | PCI commonly presents with vertigo, nystagmus, and imbalance rather than benign vestibular disease. | Supports dizziness as a clinically meaningful indicator of posterior circulation involvement. |
| **Choi and Kim, 2019** | PCI is a major cause of vascular vertigo, often under-recognized in older adults. | Strengthens the rationale for examining dizziness in elderly stroke patients with posterior circulation stenosis. |

Note: VB = Vertebrobasilar; PCI = Posterior Circulation Ischemia; TIA = Transient Ischemic Attack; mRS = modified Rankin Scale.
